# Supplementary material for: Examining recurrent hurricane exposure and psychiatric morbidity in Medicaid-insured pregnant populations
Source: PLOS Ment Health. 2024 Jun 13;1(1):e0000040. doi: 10.1371/journal.pmen.0000040 (PMC12798489; doi:10.1371/journal.pmen.0000040)
Supplement: S1 Table — (DOCX) [file pmen.0000040.s001.docx]

**S1 Table.** International Classification of Diseases, Tenth Revision, Clinical Modification (ICD-10-CM) diagnosis codes used to operationalize major and sub-categories of maternal mental disorders.

| **Major category** | **Sub-category** | **Primary and Secondary* ICD-10 codes** |
| --- | --- | --- |
| Maternal Disorders of Pregnancy (MDP) | Mental disorders complicating pregnancy or childbirth | O99.34X, O99.342, O99.343, O99.344 |
| Severe Mental Illness (SMI) | Bipolar disorder | F30.10, F30.11, F30.12, F30.13, F30.20, F30.3, F30.4, F30.8, F30.9, F31.1, F34.81, F39.X |
|  | Psychotic disorders | F06.0, F06.2, F20.89, F20.0, F20.1, F20.2, F20.5, F20.81, F20.89, F20.9, F22.X, F23.X, F24.X, F25.0, F25.8, F25.9, F28.X, F29.X, F53.1 |
| Perinatal Mood or Anxiety Disorders (PMAD) | Depression  Anxiety | F32.X, F33.X, F34.1, F41.8, F43.20, F43.21, F43.23, F43.25, F43.29, F45.89, F53.0, F06.31, F06.32  F06.4, F4.X, F68.1, F93.8, F95.0, F99, R45.7, R46.89 |
|  |  |  |
| Substance Use Disorder in pregnancy (SUDP) | Drug use complicating pregnancy, childbirth, and the puerperium | O99.32 |

*Maternal mental disorders were defined using primary and secondary ICD-10 diagnosis codes. While the primary diagnosis is intended to capture the diagnosis most serious and the secondary diagnosis as conditions that coexist at the same time of admission, there is no evidence to suggest that clinicians use these codes uniformly and even less is known about the implementation of these codes among psychiatrists and other mental health professional shortage areas.^25^ To address this uncertainty and ensure sufficient sample size for these rare outcomes, we included the primary and secondary diagnosis codes, a common approach to capture acute conditions using hospital administrate data.
