# Supplementary material for: Examining recurrent hurricane exposure and psychiatric morbidity in Medicaid-insured pregnant populations
Source: PLOS Ment Health. 2024 Jun 13;1(1):e0000040. doi: 10.1371/journal.pmen.0000040 (PMC12798489; doi:10.1371/journal.pmen.0000040)
Supplement: S2 Table — (DOCX) [file pmen.0000040.s002.docx]

**S2 Table.** Hurricane exposure status of a retrospective cohort study of eligible pregnant Medicaid beneficiaries among North Carolina residents, 2015-2020.

| **Matthew (2016)** | | **Florence (2018)** | | **Michael (2018)** | | **Dorian (2019)** | |
| --- | --- | --- | --- | --- | --- | --- | --- |
| Exposed  n (%) | Unexposed  n (%) | Exposed  n (%) | Unexposed  n (%) | Exposed  n (%) | Unexposed  n (%) | Exposed  n (%) | Unexposed  n (%) |
| 34418 (14.41) | 204483 (85.59) | 90069  (37.70) | 148832 (62.30) | 5158  (2.16) | 233743 (97.84) | 40466 (16.94) | 198435 (83.06) |
