# Supplementary material for: Examining recurrent hurricane exposure and psychiatric morbidity in Medicaid-insured pregnant populations
Source: PLOS Ment Health. 2024 Jun 13;1(1):e0000040. doi: 10.1371/journal.pmen.0000040 (PMC12798489; doi:10.1371/journal.pmen.0000040)
Supplement: Attachment — Submitted filename: Response to Reviewers.docx [file pmen.0000040.s003.docx]

**Response to Reviewers**

**Due April 26, 2024**

PMEN-D-24-00067

Examining recurrent hurricane exposure and psychiatric morbidity in Medicaid-insured pregnant populations

*PLOS Mental Health*

**Overall Response from the Author Team:** We are very grateful to the editor and the reviewers for the opportunity to revise our submission. We believe that the reviewers’ comments and suggestions have helped us achieve a much-improved manuscript resubmission that will be of great interest to *PLOS MENTAL HEALTH* readers. Below, we have included individual responses to all reviewer comments in bold.

**Academic Editor**
PLOS Mental Health

Journal Requirements:

1. Please amend your detailed online Financial Disclosure statement. This is published with the article. It must therefore be completed in full sentences and contain the exact wording you wish to be published.

**Response AE1: Our financial disclosure statement has been amended.**

a) State the initials, alongside each funding source, of each author to receive each grant. For example: "This work was supported by the National Institutes of Health (1R03ES031228-01A1 to JR, MS) and the National Oceanic and Atmospheric Administration (NA21OAR4310312 to JR, NM, CR; NA19NES4320002 to JR, KH, CS, TW)."

**Response AE1.a: The section on funding sources has now been revised.**

b) State what role the funders took in the study. If the funders had no role in your study, please state: “The funders had no role in study design, data collection and analysis, decision to publish, or preparation of the manuscript.”

**Response AE1.b: We now state that the following on the role of the funders:** “The funders had no role in study design, data collection and analysis, decision to publish, or preparation of the manuscript.”

2. Please update your online Competing Interests statement. If you have no competing interests to declare, please state: “The authors have declared that no competing interests exist.”

**Response AE2: Our Competing Interests statement now states: “The authors have declared that no competing interests exist.”**

3. Please provide separate figure files in .tif or .eps format only and ensure that all files are under our size limit of 10MB.

For more information about how to convert your figure files please see our guidelines: <https://journals.plos.org/complexsystems/s/figures>

**Response AE3: Figures are now included as a .tif file format and are under the size limit of 10MB.**

4. Please include a separate legend for Figure 1 in your manuscript.

**Response AE4: Figure 1 has been revised to include a separate legend in the manuscript.**

5. We have noticed that you have uploaded Supporting Information files, but you have not included a list of legends. Please add a full list of legends for your Supporting Information files after the references list.

**Response AE5: A full list of legends for Supporting Information files is now included after the reference list.**

Reviewer #1: The manuscript is an analysis of mental health claims in the North Carolina Medicaid data and hurricane exposure.
In a few places it is unclear whether the comparison is non-pregnant women or pregnant women in unaffected communities.

**Response 1.1: We are grateful for the critical and thoughtful review by the reviewer. We hope the additional clarification provided below further enhances our revised manuscript.**

I am not very familiar with the DiD method, but I’m probably not the only one in your readership. I tend to think of it as being conducted at an ecologic level, but some of the description sounds more like an individual level, i.e., each person in the dataset is coded as yes/no for having this outcome. Are you modeling the number of diagnoses per county, the proportion of pregnant ED admissions with a mental health code, the number of MH diagnoses per 1000 births or pregnancies?

**Response 1.2: We thank the reviewer for this comment. The methods section has been revised to reflect the above text:**

The DID method is an econometric approach that helps answer the question, what would have happened to maternal mental disorders in our study, if the intervention (i.e., hurricanes) had not taken place. The DID is a quasi-experimental methodology that estimates the true impact of the intervention (e.g., hurricane). In this study, we analyzed repeated cross-sectional data that followed different groups of pregnant populations residing in hurricane-impacted counties and control or non-impacted counties one year before and one year after each individual hurricane. Each county served as its own control and consists of repeated observations of maternal mental health-related ED visits for individual counties; whereby the assumption is that county-level psychiatric morbidity in a given county before the hurricane serves as a counterfactual for what county-level psychiatric morbidity in pregnant populations would have been in the absence of a given hurricane event. In the DID methodology, the impacted group is exposed to a hurricane (i.e., intervention) and the control group is not exposed to the intervention. The DID design then compares the changes within each group (intervention versus control) between the pre-treatment (i.e., the first difference) and post-treatment (i.e., the second difference) periods. A DID estimator is then computed to compare the differences between the intervention and control group post-hurricane (second difference), while accounting for the existing differences between intervention and control groups pre-event (first difference). A key advantage of the DID or quasi-experimental design is that the assignment of treatment or control group simulates that of a randomized control trial and with the addition of the propensity score weight, controls for unobserved and observed confounders that are constant over time, resulting in balanced treatment and control groups.

Given that diagnosis of a mental health issue among pregnant Medicaid patients in an ED relies on several levels of conditions, it would be helpful to explain why the trends can be attributed to the hurricanes and not other factors, which might vary by hurricane status.
- Providers may be more likely to diagnose mental health issues after a disaster
- There may be fewer qualified providers after a disaster
- People may use EDs more if primary care clinics are shut down. Similarly, the higher risk in the maternity care deserts may indicate lack of other care options, not a higher rate of the outcome.
- EDs may be closed due to the disaster
- People may be dealing with disaster aftermath and be less likely to seek medical care for less serious problems
- Medicaid coverage may be extended or reduced (most pregnant women are eligible, but eligibility criteria and requirements are often changed after a disaster)
- The overall population in the county may have changed

**Response 1.2:We agree that the reviewer has raised a number of alternative explanations behind the increasing trends in ED-visits for some maternal mental disorders post-storm. Please refer to Response 1.1 on the benefits of the DID design to infer causality. Unfortunately, given the limitations of administrative data on emergency department visits, we are unable to address or measure many of these potential explanations. We have added mention of these factors in our limitations section, given we don’t have data on provider-level changes in diagnosing maternal mental disorders or availability, etc.**

- The comparison group is not always clear to me. If someone does not have the outcome, are they are pregnant case visiting the ED for another reason? If so, couldn’t changes in other outcomes also change the relative proportion visiting for pregnancy/MH?

**Response 1.3: We thank the reviewer for this comment and have added additional details on the comparison group in our Methods section.**

The method of defining storm exposure is interesting. How much does it vary from other options, like disaster declarations or wind speed? Since it’s based on crop loss, is there any possibility that the level of agricultural activity in a county would cause variation in exposure definition.

**Response 1.4: We thank the reviewer for this inquiry on how well our metric for exposure compares with other options. As mentioned in the methods section, our HIP-WI metric is unique in that it does include meteorological components, including wind speed and total rainfall derived from NOAA’s National Hurricane Center. We decided not to compare HIP-WI with FEMA disaster declarations, given the highly political nature in which FEMA county disaster declarations are determined (Salkowe and Chakraborty, 2009, Gasper 2015, Husted and Nickerson, 2013, Reeves 2011, Dynes and Huber 2015, Daniels 2013cite). For instance, states politically important to the president have a higher rate of FEMA federal disaster declaration status, and disaster expenditures are higher in states having congressional representation on FEMA oversight committees (Garrett and Sobel 2003).**

**- Salkowe, R. S., & Chakraborty, J. (2009). Federal Disaster Relief in the U.S.: The Role of Political Partisanship and Preference in Presidential Disaster Declarations and Turndowns. Journal of Homeland Security and Emergency Management. 6(1).** [**https://doi.org/10.2202/1547-7355.1562**](https://doi.org/10.2202/1547-7355.1562)

**-Gasper, J. T. (2015). The Politics of Denying Aid: An Analysis of Disaster Declaration Turndowns. Journal of Public Management & Social Policy. 22(7).** [**https://digitalscholarship.tsu.edu/cgi/viewcontent.cgi?article=1027&context=jpmsp**](https://digitalscholarship.tsu.edu/cgi/viewcontent.cgi?article=1027&context=jpmsp)

**-Husted, T., & Nickerson, D. (2013). Political Economy of Presidential Disaster Declarations and Federal Disaster Assistance. Public Finance Review, 41(1), 35-37.** [**https://doi.org/10.1177/1091142113496131**](https://doi.org/10.1177/1091142113496131)

**-Reeves, A. (2011). Political Disaster: Unilateral Powers, Electoral Incentives, and Presidential Disaster Declarations. The Journal of Politics, 73(4), 1142-1151.** [**https://doi.org/10.1017/s0022381611000843**](https://doi.org/10.1017/s0022381611000843)

**Dynes, A. M., & Huber, G. A. (2015). Partisanship and the allocation of federal spending: Do same-party legislators or voters benefit from shared party affiliation with the president and House majority? American Political Science Review, 109(1), 172–186.** [**https://doi.org/10.1017/s000305541400063x**](https://doi.org/10.1017/s000305541400063x)**\**

**Daniels, R. S. (2013). The rise of politics and the decline of vulnerability as criteria in disaster decisions of the United States, 1953–2009. Disasters, 37(4), 669–694.** [**https://doi.org/10.1111/disa.12026**](https://doi.org/10.1111/disa.12026)

**Garrett, T. A., & Sobel, R. S. (2003). The political economy of FEMA disaster payments. *Economic inquiry*, *41*(3), 496-509.**

**Please also note that in the manuscript, we provide additional detail on the HIP-WI metric:**

The HIP-WI is a supplemental crop insurance program intended to provide coverage to agricultural workers in counties exposed to sustained tropical storm-strength winds, limited to named storms [25]. Wind extent is determined by meteorological variables derived from the International Best Track Archive for Climate Stewardship (IBTrACS) data from the National Oceanic and Atmospheric Administration’s National Hurricane Center. To issue a county loss trigger: a) a hurricane must have a maximum sustained surface winds of 64 knots (74 mph) or greater, or b) a tropical storm event must have been characterized by a sustained surface wind speed of 34 knots (39 mph) and at least 6 inches of total precipitation accumulating over four consecutive days (i.e., one day before the storm, the day of the storm, and two days after the storm hits). Individual convex hulls are computed based on the initial and final tropical storm points, the estimated center point, along with their corresponding oval buffers. Convex hulls that overlap with county boundaries defined by the Census Bureau indicate counties exposed to hurricanes and determine county loss triggers for reimbursement [26]. To approximate storm exposure for NC counties, we used published county loss triggers to define exposed compared to unexposed counties and total rainfall to approximate the severity of the storm.

Why are maternity care deserts coded as 3 and low access as 1?

**Response 1.5: We thank the reviewer for this comment and have ensured that the categorical coding definitions for maternity care deserts has been corrected.**

This sentence doesn’t make sense: “We opted to categorize recurrent storm exposure this way, because there were such a small number of individuals who experienced two recurrent storms.” Do they mean 2 or more?

**Response 1.6: We have edited the sentence and it now says:**

For this analysis, we operationalized recurrent storm exposure as county-level experience of multiple hurricane events (e.g., Matthew, Florence, Michael, or Dorian) in the study period. Counties were flagged as being “exposed” to a given storm if a loss trigger was flagged as identified by the HIP-WI and “control” counties were assigned based on the absence of a loss trigger. Possible values of the cumulative exposure variable ranged between 0 and 4. The final recurrent storm variable consisted of the following categories: 0 = no storm exposure, 1 = exposure to one storm, and 2 = exposure to two or more storms. We opted to categorize recurrent storm exposure in this way because there were so few individuals who experienced two recurrent storms only. Therefore, to ensure an adequate sample size, we combined a total of 2 or 3 storms experienced into the ‘2 or more storms’ category.

The definition of recurrent exposure is confusing. My understanding is that it is just whether a woman goes to an ED in that county, and the county’s level of hurricane is the exposure, not the individual’s. Since individuals are not linked over time, presumably you don’t know when someone moved into the county? Was gestational age used to determine whether the storm had occurred during the pregnancy?

**Response 1.7: We thank the reviewer for drawing our attention to the need for enhanced clarity on how recurrent storm exposure was operationalized. In the manuscript, we state:**

In this paper, we conceptualize recurrent exposure as the repeat occurrence of a climate-induced hurricane in the same geographic location, recurring typically on an annual basis.

Counties that experienced loss triggers as identified by the HIP-WI for a given storm were considered ‘exposed’ and assigned to the treatment group. Counties that do not experience loss triggers disaster for a given storm were assigned to the unexposed or control group.

**However, we recognize that we can be more explicit on how we then operationalized recurrent storm exposure in the cumulative model section of the grant. The revised text can be found in Response 1.6 above.**

Substance use as a proxy for IPV seems like a stretch; it may be sometimes be the case, but people also use substances to cope with stress and that seems like a more direct hypothesis.

**Response 1.8: We thank the reviewer for this comment. We have included additional context around how an increase in substance use may be a coping mechanism for disaster-related stress. However, we’d also like to note that we say it may be that substance use serves as a proxy for IPV, but more research is needed. See edits to the text below in red:**

While we observed that a single hurricane event was not associated with an increased risk of psychological morbidity during pregnancy, with the exception of elevated risk of substance use, very few studies have established a connection between direct disaster exposure and increased substance use, as a coping mechanism for disaster-related stress, in pregnant populations [55]. However, some literature has drawn an important connection between a mirrored increase in intimate partner violence and substance use for women who have experienced a natural disaster [56]. The majority of the disaster literature has not uncovered a substantial increase in substance use disorders post-event [40,57]. While, it may be that substance use serves as a proxy for intimate partner violence, an issue that many providers have limited experience with [58], more research is needed to explore the linkages between substance use, disasters, and intimate partner violence.

The authors say they can’t take into account mass shootings and the like, but it seems like those could be incorporated into the analysis, if they wished.

**Response 1.9: We thank the reviewer for this thoughtful suggestion; however, please note that the influence of mass shootings is not the manuscript's primary or secondary focus. It’s important to note that the DID analysis does remove biases in the post-storm period comparisons between the treatment and control groups that may be the results of a permanent difference between the two groups in addition to biases from comparisons over time in the treatment or storm-impacted group that might be the result of trends due to other causes of the outcome (e.g., mass shootings).**

Reviewer #2: This article uses indirect sources to make inferences (Medicade records, federal funding in the counties etc.)about mental health of pregnant women who are exposed to multiple disasters. Overall the measures are analyzed and articulated appropriately. The many limitations to using these indirect sources was well described.

**Response 2.1: We are grateful for the thoughtful critique you provided and additional questions to consider to enhance the overall clarity of this work.**

Unclear areas that could be made further explicit in the study, if possible, are:

(1) What was the degree of danger women in these areas were at risk for as the respective storms passed though eastern NC?

**Response 2.2: Our study population includes women from a hospital administrative dataset, and the individual’s direct experience of a storm cannot be determined. Instead, we use a county-level metric to approximate hurricane exposure. We have added this limitation to our Discussion section.**

What was downgraded intensity and degree of flooding and infrastructure impact?

**Response 2.3: We thank the reviewer for this question. However, please note that our hurricane exposure metric was not intended to measure downgraded intensity, but total rainfall was factored into a county loss trigger (see Response 1.4).**

There are degrees of "exposure" if you can describe more. How long did the hurricane systems affect the targeted affected communities?

**Response 2.4: We thank the reviewer for this comment. Unfortunately, we were unable to measure the degrees of exposure using the HIP-WI metric. However, a key advantage of this metric is that it considers meteorologic based parameters, wind extent, and total precipitation, parameters used in prior studies to characterize hurricane exposure (Grabich et al. 2016). The dates of each storm are included in Figure 1. However, we have updated the language in the *Limitations* section to address this point:**

An additional limitation is that storm exposure was approximated using wind speed and total precipitation at the county-level, and data on individual-level direct experience or varying levels of storm severity across a county were not captured.

Grabich, S. C., Horney, J., Konrad, C., & Lobdell, D. T. (2016). Measuring the storm: Methods of quantifying hurricane exposure with pregnancy outcomes. *Natural Hazards Review*, *17*(1), 06015002.

For multiple exposures, women were only pregnant for one of the exposures, right, or both? Were they pregnant when two different systems went through respective dates? That was not clear.

**Response 2.5: We thank the reviewer for this helpful comment. In the paper we state that “Given the cross-sectional nature of our ED data, we were unable to longitudinally track pregnant persons over time, but we were able to assess ecological changes in ED utilization for each county over time.” However, we’ve gone back through the methods section to make explicit that only counties and not pregnant cases were longitudinally monitored over time. Please also see *Response to Reviewer 1.6 & 1.7.***
